# On the Slow-Time *k*-Space and its Augmentation in Doppler Radar Tomography

**DOI:** 10.3390/s20020513

**Published:** 2020-01-16

**Authors:** Hai-Tan Tran, Emma Heading, Brian W.-H. Ng

**Affiliations:** 1 Intelligence, Surveillance, and Space Division, Defence Science and Technology Group, Edinburgh, SA 5111, Australia; emma.heading@dst.defence.gov.au; 2 Faculty of Engineering Computer & Mathematical Sciences, School of Electrical and Electronic Engineering, The University of Adelaide, North Terrace Campus, Adelaide, SA 5005, Australia; brian.ng@adelaide.edu.au

**Keywords:** slow-time *k*-space, spatial frequency, Doppler radar tomography, radar imaging, *k*-space augmentation, high-resolution narrowband radar

## Abstract

Doppler Radar Tomography (DRT) relies on spatial diversity from rotational motion of a target rather than spectral diversity from wide bandwidth signals. The slow-time *k*-space is a novel form of the spatial frequency space generated by the relative rotational motion of a target at a single radar frequency, which can be exploited for high-resolution target imaging by a narrowband radar with Doppler tomographic signal processing. This paper builds on a previously published work and demonstrates, with real experimental data, a unique and interesting characteristic of the slow-time *k*-space: it can be augmented and significantly enhance imaging resolution by signal processing. High resolution can reveal finer details in the image, providing more information to identify unknown targets detected by the radar.

## 1. Introduction

Tomography is a general imaging technique that is based on lower-dimensional projections of an object from different spatial aspects, which are then processed using the projection-slice theorem [1] to reconstruct an image of the object. Radar tomography uses reflective scattering phenomenology and radar waveforms for the measurements, which may be wideband or narrowband. Wideband waveforms exploit *spectral* diversity as system resources to facilitate radar imaging and have probably been the most exploited resources in practical applications in the last few decades. The well-known synthetic aperture radar (SAR) and inverse SAR (ISAR) imaging techniques may be described as two special forms of wideband tomography, in which another system resource—*spatial* diversity—is exploited only minimally [2]. Range-Doppler ISAR imaging, and stripmap SAR in particular, typically involve aspect angle changes of a few degrees [3,4,5]. This constraint of small rotation angles in the linear phase regimes allows the image inversion processing to take advantage of the computationally efficient fast Fourier transform (FFT) without needing signal interpolation onto rectangular grids.

Spotlight SAR makes use of wider angles [6], while circular SAR [7] may coherently process up to a complete cycle of target aspect rotation, with sophisticated and precise motion compensation in range. More notably, in the associated spatial frequency spaces, also known as *k*-spaces [2], traditionally intensive interpolation processing prior to image inversion processing may be necessary. Nevertheless, these forms of SAR and ISAR rely on the bandwidth resource to achieve high down-range resolution, and so can be considered as belonging to the category of ‘wideband radar tomography’.

Radar tomographic imaging with ultra-narrowband or single-frequency waveforms relies on spatial diversity as the only system resource for image formation [8,9,10,11]. Spatial diversity may be realized by: (i) having a radar with multiple receivers looking at the target from diverse angular locations, the received signals from which are processed coherently, or (ii) using a single receiver looking at a target undergoing relative rotational motion, i.e., changing target aspect. Both cases widen the angular extents of the measurement support of the received signal in the *k*-spaces.

Previous work [2] showed that narrowband radar tomography can be most effectively formulated in the *slow-time* *k*-space in conjunction with the classical Doppler processing and Doppler radar tomography (DRT) [12,13]. The DRT algorithm applies the projection-slice theorem in which the inputs of the target’s cross-range projections are formed from Doppler profiles. The slow-time *k*-space is not only convenient for describing the DRT algorithm, it is also a natural tool to formulate high-resolution DRT imaging with an *augmentation* of its measurement support. Augmentation is the process of significantly enlarging the support of the slow-time *k*-space by using longer coherent processing intervals (CPI) in the DRT algorithm and correcting for nonlinear phase effects due to strong rotational motion. This ‘augmentability’ is a unique characteristic of the slow-time *k*-space.

The introduction of nonlinear phase terms in the *k*-space augmentation causes a blurring effect in the resulting image. Spectral compression techniques for chirped signals can be used to address this problem using bilinear transforms such as the Wigner-Ville Distribution (WVD), the Cohen’s class and the time-frequency distribution series (TFDS) as discussed in [14]. The problem with these techniques is the presence of undesirable cross terms when instantaneous component frequencies may overlap, which is the case for DRT imaging [13]. The combination of the fractional Fourier transform and S-method was used to overcome the problem of cross terms in [13], which demonstrated the slow-time *k*-space augmentation with DRT. The current work is extended to a more novel technique based on the orthogonal matching pursuit (OMP) technique, inspired by related work in compressive sensing.

Radar imaging naturally is suited to compressive sensing techniques, given that real targets often resemble a sparse collection of discrete point scatterers [5,15]. OMP is fundamentally a technique for parameter estimation by matching a given signal to a dictionary of possible elemental functions spanning a finite parameter space. The dictionary is designed for the particular application and has been applied to the area of DRT imaging in varying contexts [16,17,18]. The particular application in this work is to estimate the non-linear phase term in the radar signal to reduce the image blurring for improved resolution. The main contribution of the paper is two-fold: to highlight the augmentability of the slow-time *k*-space as a fundamentally useful characteristic for narrowband radar imaging, and to present a novel application of the OMP technique to such augmentation processing.

The slow-time *k*-space processing technique as presented in this paper provides a complimentary approach to traditional high-resolution ISAR imaging. The dependence of wide bandwidth signals for high resolution in ISAR imaging is not always readily achievable within the confines of the available spectrum and limitations at lower frequency bands [9,19]. The proposed high-resolution imaging scheme can lead to improved target recognition despite an absence of wide bandwidth signals, provided sufficient spatial diversity is available. This is an important capability of great interest to the radar research community [20].

The rest of the paper is organized as follows. The next Section summarizes the fundamental theory: system geometry and signal model, cross-range bandwidth and resolution, and DRT. Section 3 describes the slow-time *k*-space and its augmentation with OMP processing. Section 4 describes the experimental setup using simple point scatterers on a rotating turntable with imaging results for both standard and augmented DRT processing. The final Section presents some relevant discussion points and concluding remarks.

## 2. Background

This Section defines the signal model, the fundamental concept of cross-range bandwidth and resolution, and summarizes the known theory of Doppler radar tomography (DRT) in its standard version.

### 2.1. Signal Model

**Figure 1:**
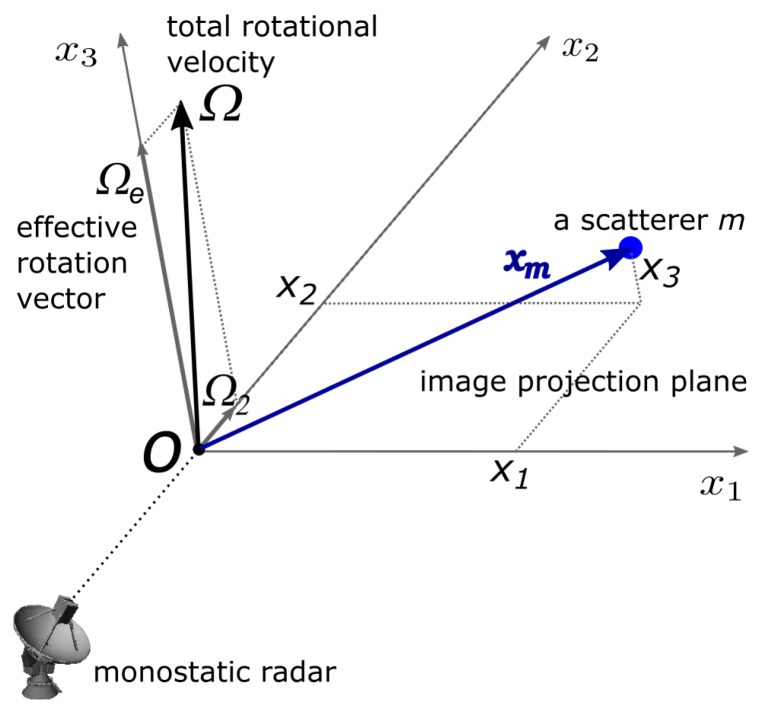
Imaging system geometry: The $\left(x_{1},x_{2},x_{3}\right)$ coordinates are defined in the local target frame, $T_{x}$. For clarity, a single point scatterer is shown at $x_{m}$, which rotates around origin *O* with velocity Ω. Targets are modeled as a discrete, distributed collection of similar point scatterers.

Consider a monostatic radar system geometry as illustrated in Figure 1. Without loss of generality, an inertial local target reference frame, denoted as $T_{x}$ with origin *O* at the target’s nominal centre of rotation, is chosen to have the the $x_{2}$ (‘down-range’) axis aligned with the radar line of sight (LOS), with $x_{1}$ axis denoting cross-range. The plane $\left(x_{1},x_{2}\right)$ is often known as the image projection plane (IPP, or just ‘image plane’). The axis orthogonal to the IPP is denoted as the $x_{3}$-axis (sometimes referred to as ‘height’). The target’s *effective* rotation vector $\Omega_{e}$ is defined as the projection of the target’s total rotational velocity vector Ω along the $x_{3}$-axis.

Using the definition above, the total rotational velocity vector can be written in $T_{x}$ as

(1) $$\Omega =(0,\Omega_{2},\Omega_{e}).$$

Physically, $\Omega_{e}$ introduces cross-range dependent Doppler shifts in the radar backscatter and is the principal reason that motion-based target imaging is possible. In comparison, $\Omega_{2}$ has minimal (sometimes deleterious) impact on radar imaging. For non-cooperative targets, neither $\Omega_{e}$ nor $\Omega_{2}$, or the orientation of the IPP itself, are known a priori. In this paper, we further assume that $\Omega_{2}=0$, and $\Omega_{e}$ is approximately constant during a coherent processing interval (CPI).

For this paper, we use an idealized point-scatterer model for the target: it is adequately modeled as an ensemble of *M* point scatterers with reflectivity coefficients $\sigma_{m}$, located in the far field of the radar. The approximate range to the $m^{\mathrm{th}}$ point-scatterer on the target with position vector $x_{m}$, defined relative to *O*, can be defined as

(2) $$R\left(x_{m}\right)\approx R\left(x_{m}\right)\cdot i_{LOS}=R_{0}+r_{m},$$

in which $R(x_{m})$ is the range vector to the $m^{\mathrm{th}}$ scatterer, $R_{0}$ is the radar range to *O*, the scatterer’s local down range is

(3) $$r_{m}=x_{m}\cdot i_{LOS},$$

and $i_{LOS}=\left(0,1,0\right)$ is the unit vector along the radar LOS in the $T_{x}$ frame.

Formulation in the $T_{x}$ frame is appropriate in traditional ISAR imaging where the change of aspect is small (a few degrees), or for signal analysis within a relatively short CPI. In contrast, radar tomography exploits spatial diversity through wide changes of target aspect. For this formulation, a second, dynamic local target frame denoted as $T_{y}$, is needed. This frame rotates with the target and coincides with $T_{x}$ at a reference time, usually assumed to be $t_{k}=0$. By the $\Omega_{2}=0$ assumption, it follows also that $T_{x}$ and $T_{y}$ share the same $x_{3}$-axis. The reason for choosing $T_{y}$ frame is that its axes are aligned with those of the underlying *k*-spaces and thus preserves angles across the $T_{y}$ frame and the *k*-spaces.

Let

$$s_{T}\left(t_{k};f\right)\propto \exp\left\{j2\pi ft_{k}\right\}$$

denote the simple transmit continuous waveform at a single frequency *f*, where only the slow time $t_{k}$ is involved; there are no pulses and hence no ‘fast time’ spanning a pulse. The slow-time index is k=0,1,2,…,K−1, and we assume a total of *K* time samples in a CPI. The received signal $s_{R}\left(t_{k};f\right)$ is a delayed version of $s_{T}\left(t_{k};f\right)$, summed over all scatterers,

(4) $$s_{R}\left(t_{k};f\right)\propto \exp\left\{-j4\pi f\frac{R_{0}\left(t_{k}\right)}{c}\right\}\;\sum_{m=1}^{M}\sigma_{m}\;\exp\left\{-j\frac{4\pi f}{c}r_{m}\left(t_{k}\right)\right\}.$$

Here, we have also assumed that radar hardware perfectly removes the carrier frequency term $\exp\{j2\pi ft_{k}\}$. The first factor in (4) describes translational motion of the target as a whole; the second factor captures the target geometry and scattering reflectivities to be processed for imaging.

Furthermore, we shall assume a linear translational motion model for the target,

(5) $$R_{0}\left(t_{k}\right)=R_{0}\left(0\right)+\nu \;t_{k},$$

where ν is the velocity, assumed known prior to DRT processing, and $R_{0}\left(0\right)$ is target range at a reference time $t_{k}=0$.

### 2.2. Cross-Range Bandwidth and Resolution

The position of each scatterer executing rotational motion with rotation vector Ω is described to a second order approximation by

$$x\left(t_{k}\right)=x_{0}+\left(\Omega \times x_{0}\right)t_{k}-\frac{1}{2}\left[\Omega^{2}x_{0}-\left(\Omega \cdot x_{0}\right)\Omega \right]t^{2},$$

where $x_{0}\equiv x\left(0\right)$ for convenience. Relative to $T_{x}$, the local down range $r_{m}$ in (4) can be expressed as

(6) $$r_{m}\left(t_{k}\right)=x_{m_{2}}+x_{m_{1}}\;\Omega_{e}\;t_{k}-\frac{1}{2}x_{m_{2}}\;\Omega_{e}^{2}\;t_{k}^{2}+\cdots ,$$

where $x_{m_{1}},x_{m_{2}}$ are the initial ($t_{k}=0$) cross range and range, respectively, of the *m*-th scatterer in $T_{x}$. The CPI duration is denoted by $T_{CPI}$. As has been thoroughly discussed in [2], although $x_{2}$ (dropping the subscript *m* for brevity) cannot be directly estimated with a zero-bandwith signal, the *first*-order term of (6) suggests that a so-called *cross-range bandwidth*,

(7) $$B_{⊥}=f\;\Omega_{e}T_{CPI}=f\;\Delta \theta ,$$

can be used to estimate cross range $x_{1}$. In other words, the target’s rotation generates an effective bandwidth which allows for the resolving cross-range measurements, as long as the rotation angle through $T_{CPI}$,

$$\Delta \theta =\Omega \;T_{CPI},$$

is sufficiently small such that higher-order terms (quadratic and above) in (6) can be ignored. In practice, the Δθ is limited to a few degrees, which is consistent with wideband ISAR imaging. Note that the presence of the (unknown) zeroth-order term $x_{m_{2}}$ means $x_{1}$ cannot be directly estimated from the time-domain signal. Doppler tomography, as formulated below, overcomes such constraints to achieve target imaging.

**Figure 2:**
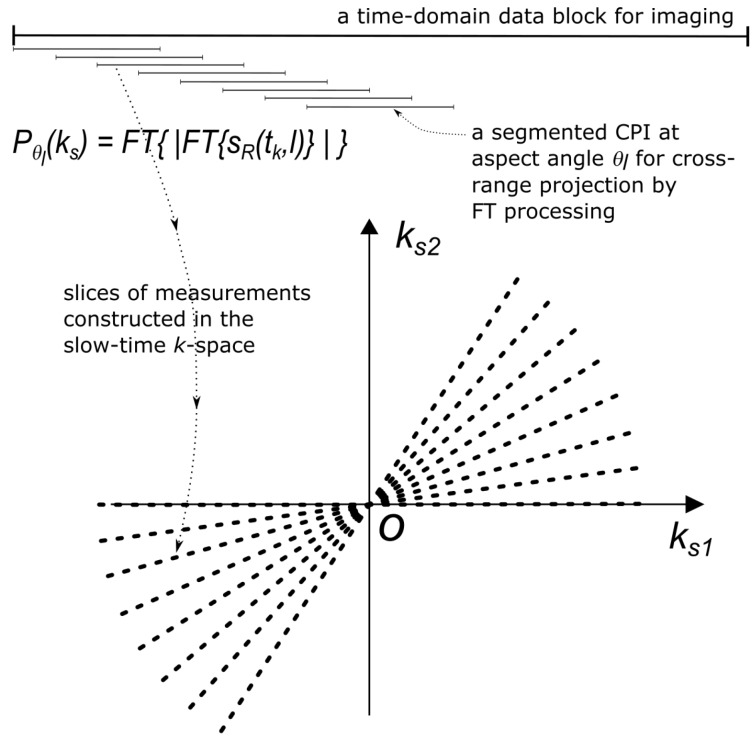
Illustration of the DRT narrowband imaging algorithm. $k_{s2}$- and $k_{s1}$ are the components of the slow-time *k*-vector $k_{s}$ aligned with the target’s initial range and cross-range directions, respectively. Each radial line represents the slow-time *k*-space samples obtained from one segmented CPI.

Consider a segmented CPI of the received signal $s_{R}\left(t_{k},l\right)$ as illustrated in Figure 2. Taking a Fourier transform over $t_{k}$ produces a Doppler profile $S_{R}\left(f_{d}\right)=F\left\{s_{R}\left(t_{k},l\right)\right\}$, with zero Doppler ($f_{d}=0$) corresponding to the centre of rotation at *O* (ignoring any residual translational motion after preprocessing). For a segment of duration $T_{CPI}$, the achievable Doppler resolution is

(8) $$\Delta f_{d}=\frac{1}{T_{CPI}}=\frac{f\;\Omega_{e}}{B_{⊥}}.$$

Each Doppler profile contains contributions from all scatterers, with the down ranges coordinates $x_{m_{2}}$ encoded as constant phase terms. Since the cross-range of a scatterer is directly proportional to its Doppler frequency $f_{d}$, namely

(9) $$x_{1}=\frac{\lambda }{2\;\Omega_{e}}\;f_{d},$$

it follows that the *magnitude* of the Doppler profile,

$$p_{\theta }\left(x\right)=\left|S_{R}\left(f_{d}\right)\right|,$$

represents a cross-range projection of the target’s reflectivity function at angle θ, the average aspect angle over the CPI. The achievable *cross-range resolution* is

(10) $$\Delta x_{1}=\frac{\lambda }{2\Omega_{e}}\;\Delta f_{d}=\frac{c}{2B_{⊥}}.$$

This expression is exactly analogous to the down-range resolution $\Delta x_{2}=c/2B$ for wideband imaging with spectral bandwidth *B*.

### 2.3. Doppler Radar Tomography (DRT)

The *Projection-Slice Theorem* (PST) states that the Fourier transform $P_{\theta }\left(f_{s⊥}\right)$ of projection $p_{\theta }\left(x\right)$ is a slice of the 2D FT of the target’s reflectivity function at aspect angle θ. This theorem can be used to invert the cross-range profiles accumulated from a range of aspect angles $\theta_{l}$ to recover the target reflectivity function, i.e., estimate the scatterer coordinates $x_{m_{1}}$ and $x_{m_{2}}$ in $T_{y}$ frame. For this to be effective, the target’s rotation must subtend a significant change in aspect angles; the 1D cross-range projections are computed in the frequency domain as discussed above, after which the target reflectivity function (image) can be reconstructed by a 2D inverse FT.

#### 2.3.1. The Monostatic DRT Algorithm

To perform radar imaging using the DRT method, it is necessary to populate the slow-time *k*-space from the radar backscatter. The algorithm to generate the slow-time *k*-space samples consists of the following steps:

- *Data segmentation:* Partition the *N* samples of the received signal $s_{R}\left(t_{n}\right)$ into *L* overlapping CPIs of *K* samples, $s_{R}\left(t_{k},l\right)$, k=0,1,…,K−1; l=1,2,…,L. These are referred to as ‘segmented CPIs’ below. Denote the overlap factor η with 0≤η<1. At the midpoint of each segment, the target aspect angle (relative to $T_{x}$) is denoted as $\theta_{l}$;
- *Translational motion compensation (TMC):* this step shifts the Doppler component induced by translational motion to zero Doppler frequency by modulating the segmented CPI by $\exp(j2\pi \nu t_{k})$, where ν is the target’s translational velocity as noted in (5). This quantity is assumed to be known or estimated by other methods. A discrete Fourier transform is then applied to the modulated segments to obtain the Doppler spectrum. The magnitude of the output,
  (11) $$p_{\theta_{l}}\left(x\right)=\left|F\{s_{R}\left(t_{k},l\right)\;\exp\left(j2\pi \nu t_{k}\right)\}\right|,$$
  is the cross-range (which is proportional to Doppler) profile for the target at an angle $\theta_{l}$ from its original orientation. Accumulate all such cross-range profiles for all the corresponding aspect angles $\theta_{l}$, i.e., for all *L* segmented CPIs.
- *Populating the k-space:* The spatial Fourier transform of $p_{\theta_{l}}\left(x\right)$
  (12) $$P_{\theta_{l}}\left(f_{s⊥}\right)=F\left\{p_{\theta_{l}}\left(x\right)\right\}$$
  at target aspect angle $\theta_{l}$ are then used as the ‘measurement samples’ in the slow-time *k*-space. As the target rotates, the measurements sweep out a region of support in slow-time *k*-space as indicated in Figure 2. Due to our choice of reference frames, the measurement population always starts close to the $k_{s1}$-axis because $p_{\theta_{1}}\left(x\right)$ is the initial cross-range profile.
- *Image inversion:* An inverse Fourier transform is applied to the populated support of the *k*-space to yield the target image. Other works have either used filtered back projection, or interpolated the samples onto a rectangular grid to utilise a standard 2D inverse Fourier transform, for this task applied [12,13]. In this paper, we use the non-uniform Fast Fourier transform (NUFFT) [21,22,23,24].

It is worth noting that the image resolution is inversely proportional to the diameter of the span of the *k*-space samples which is dependent on the cross range bandwidth $B_{⊥}$ as defined in (10). The resulting supportable size of the image is then determined from the image resolution cell multiplied by a factor of *K* being the number of samples spanning the diameter of the *k*-space. Although limited amounts of target rotation can reduce image resolution in the sparsely populated direction, here we focus on the case where a half cycle of the target scatterers is visible to the radar to completely populate the *k*-space. Under this assumption, the angular sampling density of the *k*-space samples drives the image contrast and is a trade off with computational cost [25].

#### 2.3.2. Standard DRT

By standard DRT, we refer to the case where the input cross-range profiles, as defined by (11), are Doppler migration free (DMF), and the rotation angle corresponding to each profile formed under this condition is said to be within the linear limit (of phase variation). The DMF condition can be satisfied when the segmented CPI lengths are sufficiently short such that the nonlinear phase terms in (6) are negligible and hence compensation is not necessary, or when $|x_{m}|$ is small. The former case is particularly sensitive for scatterers at larger radial distances from the centre of rotation, while the latter case applies more to scatterers sufficiently close to the centre of rotation whose Doppler frequencies are small and Doppler migration effects (if any) are also small.

As derived in the Appendix A, the standard DRT constraint on CPI rotation angle is

(13) $$\Delta \theta \leq \min\left\{\Delta \theta_{DM},\Delta \theta_{LM}\right\},\;\left(\mathrm{rad}\right)$$

where $\Delta \theta_{DM}={\left(\lambda /2\;r_{\max}\right)}^{1/2}$ is an effective rotation angle required to induce a Doppler migration (DM) of one bin, $\Delta \theta_{LM}$ is the ‘linear limit’, while DRT image resolution, in both range and cross range, is

(14) $$\Delta x_{1}=\Delta x_{2}\geq {\left(\frac{\lambda \;r_{\max}}{2}\right)}^{1/2}.\;\left(m\right)$$

Here, $r_{max}$ is the maximum radial dimension of the target. Note that Δθ and Δx are independent of rotation rate and signal sampling rate, but only on radar wavelength and the dimension of the target (through maximum radial dimension $r_{\max}$ to any scatterer). $\Delta \theta_{LM}$ is roughly 10 degrees; Equations (13) and (14) can be used as a guide to predict the expected imaging performance or applicability of standard DRT for a specific radar wavelength and target size.

The limitations imposed by these nonlinear effects at wider rotation angles can be compensated by a processing technique described in the next Section. For differentiation from standard DRT, such cases are referred to as ‘Augmented DRT’.

## 3. The Slow-Time k-Space and Its Augmentation

While it is possible to formulate the problem and solution entirely in terms of the spatial frequency space of $f_{s⊥}$, we shall keep up with tradition and formulate it in terms of a ‘*k*-space’, with

$$k_{s}=2\pi f_{s⊥}.$$

### 3.1. The Slow-Time k-Space

In basic Fourier analysis, for signal with a pulse repetition interval PRI, the Doppler frequency extent of the signal is PRF=1/PRI, which spans the interval (−PRF/2,PRF/2). Analogously, from the spatial (cross-range) resolution $\Delta x_{1}$ as given in (10), the values of spatial frequency $f_{s⊥}$ spans the interval $\left(-B_{⊥}/c,B_{⊥}/c\right)$. It follows from (7) that the interval for $k_{s}$ is

$$k_{s}\in \left(-\frac{2\pi f}{c}\Delta \theta ,\frac{2\pi f}{c}\Delta \theta \right).$$

These limits are illustrated by extents of the radial dashed lines in Figure 2. Since the cross-range profiles are computed from FFT, both the discretized time and frequency domain vectors have *K* samples. That is, the slow-time *k*-vectors $k_{s}$ corresponding to each cross-range projection contains samples given by

(15) $$k_{s}=k^{\prime }\frac{2\pi f}{c}\left(\Omega_{e}\;T_{PRI}\right)\;i_{⊥},$$

where $k^{\prime }=-K,-K+2,\ldots ,-2,0,2,\ldots ,K-2$; $T_{PRI}=T_{CPI}/K$, and $i_{⊥}$ is the cross-range unit vector (perpendicular to LOS) along the $x_{1}$-axis of the $T_{y}$ frame.

The slow-time *k*-space arises naturally out of DRT: its *radial support* determined by the cross-range bandwidth $B_{⊥}$ and its *populating samples* are $P_{\theta_{l}}\left(k_{s}\right)$ given by (12); as the target rotates, the slow-time *k*-space support is swept out in fan-like shapes around the *k*-space origin. Also, for a given $B_{⊥}$, the number of $k_{s}$ points is a processing design parameter not necessarily fixed to *K*; its chosen value however would affect only the sidelobes of the impulse response, and hence image contrast, not image resolution.

An important and useful characteristic of the slow-time *k*-space is it can be *augmented*. As implied by (7), $B_{⊥}$ can be increased by using a wider rotation angle Δθ, providing processing can effectlively correct for the nonlinear terms in the phase function of (6). In Section 3.2 below, we discuss one typical technique to correct for the *second*-order term, i.e., linear chirp components. In other words, augmentation of the *k*-space enhances resolution by permitting the CPI to be lengthened to the limit where rotational motion of all point scatterers can be modelled as linear chirps.

**Figure 3:**
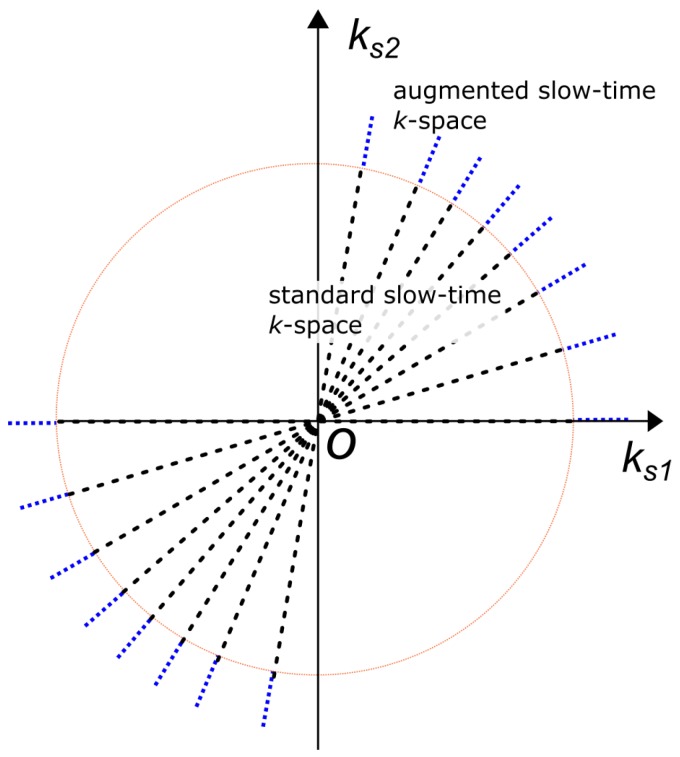
An illustration of the augmentation of the slow-time *k*-space to generate longer segmented CPIs for cross-range profile formation, which compensates for nonlinear effects of rotation arising from wider angles. The circle indicates the boundary of support in standard DRT imaging.

By comparing a standard CPI $T_{CPI}^{\left(s\right)}$ and corresponding rotation angle $\Delta \theta^{\left(s\right)}$ in the conventional linear limit of narrowband imaging to a longer CPI we define an ‘augmentation factor’

(16) $$\kappa =\frac{\Delta \theta }{\Delta \theta^{\left(s\right)}}=\frac{T_{CPI}}{T_{CPI}^{\left(s\right)}},$$

where $T_{CPI}$ is the lengthened CPI and corresponding larger rotation angle Δθ. The augmentation factor of κ describes the expansion of the cross range bandwidth $B_{⊥}$ or equivalently the radial span of the slow-time *k*-space described in (7) and (15). The DRT image resolution is inversely proportional to $B_{⊥}$ defined in (9), hence, an improvement in resolution can be achieved with adequate compensation of the linear chirps which is described further in Section 3.2. The concept of the slow-time *k*-space is illustrated in Figure 3. The DRT algorithm based on an augmented *k*-space is called augmented DRT.

### 3.2. Augmented DRT with Orthogonal Matching Pursuit (OMP)

This technique shares the same objective as the FrFTS-based technique [13] but instead makes use of a popular tool in the more modern approach of sparse signal approximation, OMP. Again, TMC is assumed to have been perfectly processed prior to this processing.

#### 3.2.1. Sparse Representation

With reference to (4) and (6), the segmented CPI signal received is represented in vector form as

(17) $$s_{R}=\Psi \sigma +\epsilon ,$$

where Ψ is the dictionary matrix of size $K\times N_{\sigma }$; σ is a length-$N_{\sigma }$ column vector of (complex-valued) atom coefficients; and ϵ is a length-*K* column vector of noise and/or clutter components. The columns of Ψ are the *chirp atoms*, of the form

(18) $$g\left(k\right)=\exp\left\{-j2\pi \left(f_{g}t_{k}+\frac{1}{2}c_{g}t_{k}^{2}\right)\right\},$$

where k=0,1,…,K−1, and the parameters

(19) $$f_{g}=\frac{2\;x_{1}\;\Omega_{e}}{\lambda },\;\mathrm{and}\;c_{g}=-\frac{2\;x_{2}\;\Omega_{e}^{2}}{\lambda }$$

respectively represent the Doppler frequency and chirp rate of a scatterer due to rotation, at reference time $t_{k}=0$ of the current segmented CPI, which define the atom $g(t_{k})$. Furthermore, let $f_{g}$ and $c_{g}$, or equivalently $x_{1}$ and $x_{2}$, be discretized as vectors of expected or possible values, of lengths $N_{f}$ and $N_{c}$ respectively, then $N_{\sigma }=N_{f}N_{c}$.

Different options for discretizing $\left(f_{g},c_{g}\right)$ lead to different definitions of the dictionary Ψ. The above option in terms of $\left(x_{1},x_{2}\right)$ uses rectangular scatterer coordinates. Another option is by polar coordinates (d,α) with

(20) $$x_{1}=d\cos\left(\alpha \right),\;x_{2}=d\sin\left(\alpha \right),$$

which may be useful when prior knowledge about the expected scatterer locations is available. It is desirable to use a coordinate grid for $\left(f_{g},c_{g}\right)$ in such a way that the grid points efficiently spans the target while keeping the total number of grid points (the dictionary size) to a minimum. A demonstration of these options is shown in Section 4.2.4.

The OMP algorithm itself is well-known, hence will not be described here (see [26] for example). In fact, OMP is only one of several sparse approximation techniques that could be used in this algorithm.

#### 3.2.2. The OMP-Based Augmented DRT Algorithm

The augmented DRT algorithm is modified from standard DRT by simply lengthening the segmented (and overlapping) CPIs with an augmentation factor κ, as defined by (16); the target signal in each CPI can then be represented as a sum of linear chirp components. The aim is then to estimate such a representation and to correct for the chirps, i.e., focusing the range profile, before applying them to remaining steps of the DRT algorithm.

For each augmented CPI, suppose the output of the OMP processing is a (sparse) representation $\left\{f_{g}^{\left(m\right)},c_{g}^{\left(m\right)}\right\}$ of size *M* with corresponding atoms $\left\{g_{m}\left(t_{k}\right)\right\}$ and coefficients $\left\{\sigma_{m}\right\}$, then a *dechirped* version for the segmented receive signal is

(21) $${\tilde{s}}_{R}\left(t_{k}\right)=\sum_{m=1}^{M}\sigma_{m}\;g_{m}\left(t_{k}\right)\to \sum_{m=1}^{M}\sigma_{m}\;{\tilde{g}}_{m}\left(t_{k}\right).$$

The right arrow → above denotes a *replacement* of the $g_{m}\left(t_{k}\right)$ atom with a corresponding *monotone* signal

(22) $${\tilde{g}}_{m}\left(t_{k}\right)=\exp\left\{-j2\pi \;f_{g}^{\left(\mathrm{mid}\right)}t_{k}\right\},$$

with Doppler frequency

(23) $$f_{g}^{\left(mid\right)}=f_{g}^{\left(m\right)}+\frac{1}{2}\;c_{g}^{\left(m\right)}t_{\mathrm{mid}},$$

so defined as the instantaneous frequency at $t_{\mathrm{mid}}$–the middle time of the segmented CPI. The operation

(24) $$p_{\theta_{l}}\left(x\right)=\left|F\{{\tilde{s}}_{R}\left(t_{k}\right)\}\right|$$

then would give a focused cross-range projection for tomographic processing.

The augmentation algorithm, applied to each CPI of the augmented DRT algorithm, can thus be summarized as follows.

0. *Initialize:*
  − define or select expected intervals of Doppler frequency $f_{g}$ and chirp rate $c_{g}$;
  − define the corresponding chirp atoms and set up the dictionary Ψ;
  − input segmented CPI data $s_{R}\left(t_{k}\right)$;
1. Compute the OMP-based sparse solution;
2. Replace all chirp atoms in the sparse solution with single-tone sinusoid functions with Doppler frequency at the mid-point of the segmented CPI;
3. Compute the focused cross-range profile $p_{\theta_{l}}$ as given by (24).
4. Compute NUFFT on the populated slow-time *k* space to produce the output image.

## 4. Experimental Results

We present two different datasets using simple point-like scatterers on a rotating turntable to represent a target. This scenario is analagous to rotating components on a target such as a helicopter rotor blade tips [20,25,27]. The first dataset is a target with a small dimension and small scatterers to showcase improvements in resolution. The second dataset is representative of a much larger target which highlights the effect of blurring in the image that we aim to remove for improved image resolution.

### 4.1. Small Target

#### 4.1.1. Experimental Setup

The data was collected in the Mumma Radar Laboratory at the University of Dayton, Ohio, USA. Although the aim of the study is narrowband imaging, a wideband waveform at X-band was used with stepped-frequency pulses between 8 GHz and 12 GHz, over 101 regular frequency steps. Only the measured data from one of the available discrete frequencies $f_{k}$ was used to study narrowband tomographic radar imaging.

**Figure 4:**
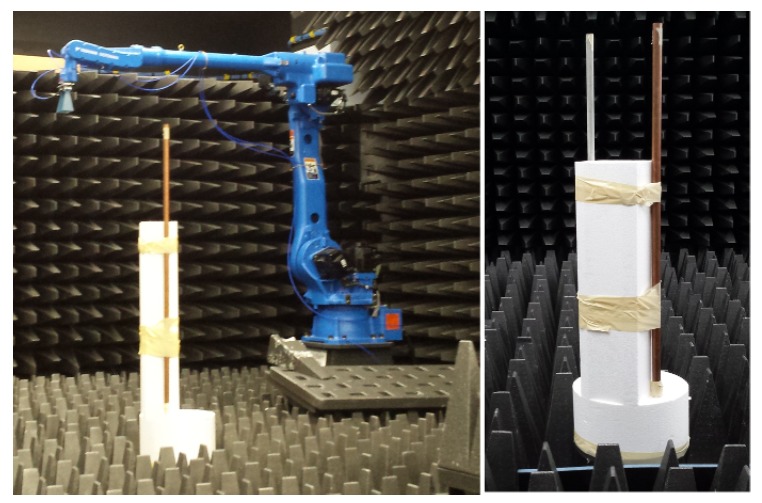
An example of an antenna mounted on a robotic arm at the Mumma Radar Laboratory with the two vertical metallic rods secured to the rotating pedestal.

The transmit and receive horn antennas were mounted on separate robotic arms which could be oriented and positioned with high precision. The measurements were conducted in a controlled laboratory environment with some Radar Absorbing Material (RAM) reducing the radar reflections from the floor and walls. The experimental target consisted of two vertical metallic rods, separated by 19 cm (approximately), emulating two point scatterers which rotated around a vertical pedestal, as illustrated in Figure 4. The maximum radial distance is 11 cm. The antennas were kept stationary whilst the target was rotated through $360^{\circ }$, at $0.1{}^{\circ }$ steps. At each step, the stepped-frequency waveform was transmitted and sampled, one sample for each frequency.

#### 4.1.2. System Requirements

Successful imaging is not dependent on shifts in relative velocity of the target from pulse to pulse, in fact the target could completely stop at each sampled rotation angle [28]. This is the case when a target rotates on a turntable with a very slow rotation rate during which the Doppler frequency is derived from the change in phase in time from the different target perspectives. Therefore we describe the system parameters such as target rotational speed and radar sampling rate based on the angular sampling rate.

While the full theoretical details are included in the Appendix A, the key requirements are summarized as follows.

- $f_{k}$: we choose the lowest and highest frequencies available in this experiment, 8 and 12 GHz, corresponding to λ=3.75 or 2.5 cm. With $r_{\max}\approx 0.11$ m, $\Delta \theta_{DM}\approx 23.7{}^{\circ }$ or $19.3{}^{\circ }$, respectively. We also choose $\Delta \theta_{LM}=10^{\circ }$; the system is thus $\Delta \theta_{LM}$-limited and poor imaging performance can be expected from standard DRT;
- Inequality (A1) is the Doppler ambiguity free condition; PRF should be designed such that the angular sampling rate $PRF_{a}=PRF/\omega$ (in samp/rad) is greater than $\left(4\;r_{\max}/\lambda \right)$ (11.7 or 17.6 for this setup), but with as small a margin as possible, to ease hardware requirement.
- The angular sampling interval of $0.1{}^{\circ }$ per sample in the experiment translates to a $PRF_{a}=573$ samp/rad. Over the chosen $\Delta \theta_{LM}$ value, 100 samples are available. To reduce computational cost while retaining a reasonable FFT length and satisfying the Doppler ambiguity free condition, we use a *down sampling* ratio of 3:1, leading to K=33 samples per CPI, and $PRF_{a}\approx 191$ (samp/rad). This choice also automatically satisfies the constraint in (A8).

Realistic values for PRF and ω can also be chosen such that PRF/ω=191, however, this is not necessary for DRT processing.

For each of the selected frequencies, an elliptic filter with a very narrow stop band is also applied to the signal as a pre-processing for clutter removal. Results are shown in Section 4.2.3 and Section 4.2.4.

#### 4.1.3. Standard DRT Imaging

**Figure 5:**
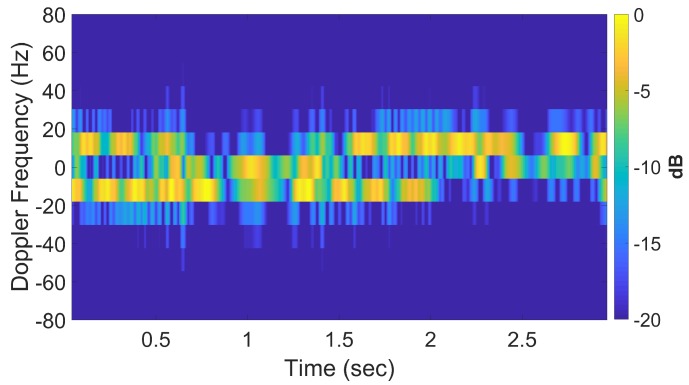
Spectrogram using standard DRT processing for f=8 GHz.

**Figure 6:**
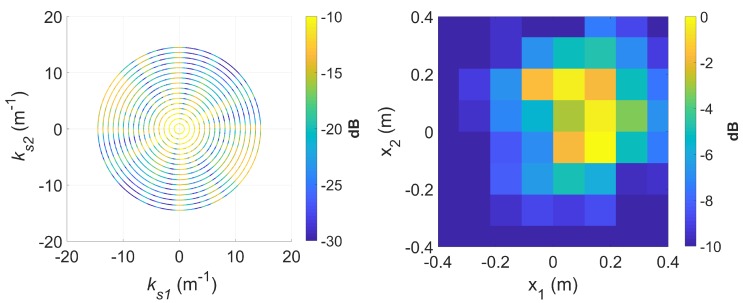
The slow-time *k*-space support (left) and corresponding standard DRT image (right), at f=8 GHz.

To demonstrate *k*-space augmentation and the usefulness of sparse signal approximation, some typical results of standard DRT imaging is now shown. Figure 5 shows a spectrogram of the signal at 8 GHz and Figure 6 shows the corresponding slow-time *k*-space support and standard DRT image. We have used an overlapping factor η of 0.99 in the segmentation step to provide a very smooth angular coverage of the *k*-space. However, standard DRT imaging performance is poor; the *k*-space support is small; the two scatterers (metallic rods) are not distinguishable in the image.

**Figure 7:**
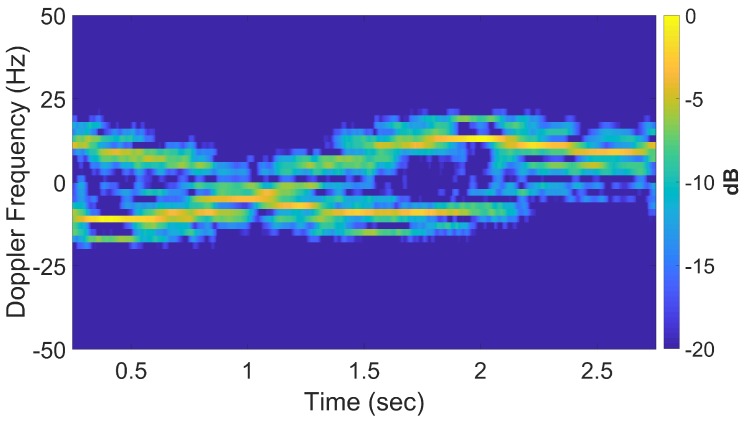
Signal spectrogram with augmented CPIs, κ=6, at f=8 GHz.

**Figure 8:**
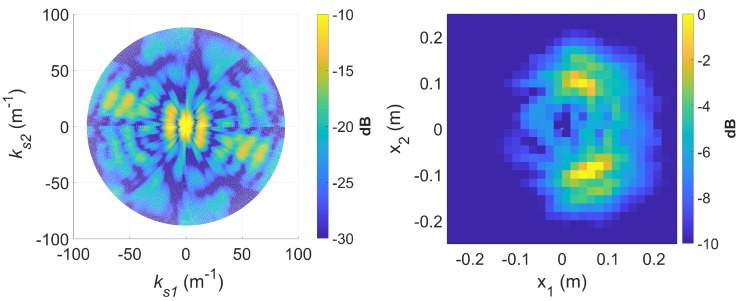
The slow-time *k*-space support (left) and image (right) for standard DRT with κ=6, at f=8 GHz.

If longer CPIs are used with the standard DRT algorithms, image blurring occurs. Suppose κ as defined by (16) is set to 6, the Doppler resolution in the spectrogram of the signal becomes higher, as shown in Figure 7. When the corresponding cross-range profiles (with Doppler bin migration effects present) are applied to standard DRT, the resulting image is in Figure 8.

**Figure 9:**
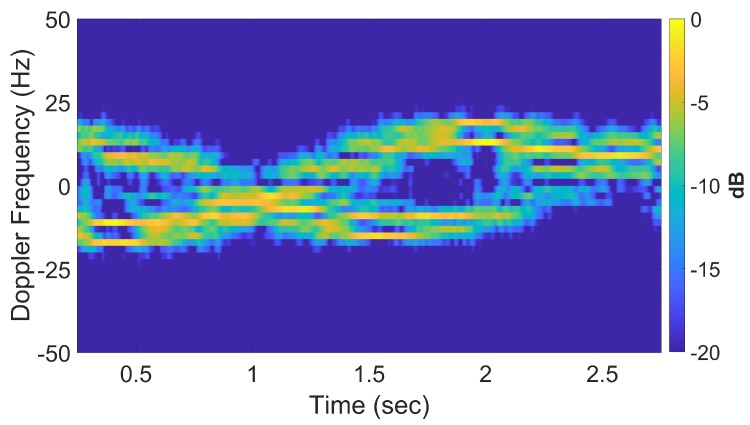
Signal spectrogram with augmented CPIs, κ=6, at f=12 GHz.

**Figure 10:**
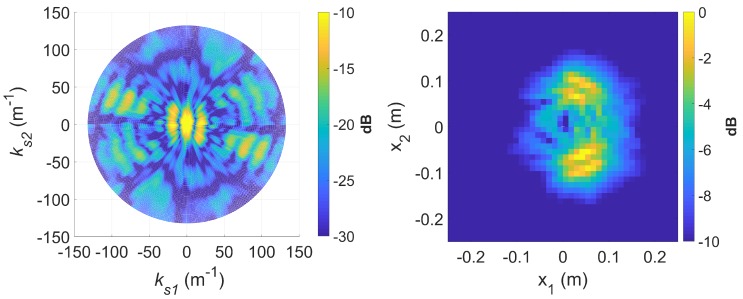
The slow-time *k*-space support (left) and image (right) for standard DRT with κ=6, at f=12 GHz.

For a better insight into the electromagnetic scattering effects in this experiment, similar results using the highest frequency (12 GHz) available are shown in Figure 9 and Figure 10. From Figure 7 and Figure 9 it is clear that in addition to direct (specular) scattering off the inner side of a metallic rod, creeping waves around the rods are the most likely cause of the twin sinusoidal traces for each of the rods [29]. The effects are more pronounced with the shorter wavelength of 2.5 cm, which is more comparable to the rod diameter of approximately 2 cm. The double scattering effects are highlighted in the DRT image.

Note that image blurring in standard DRT imaging is only in the azimuthal direction; image focusing is still generally achieved in the radial direction.

#### 4.1.4. Augmented DRT Imaging with OMP

To apply OMP for image focusing, the dictionary Ψ is set up with chirp atoms as defined in (18) and (19). As mentioned in Section 3.2.1, two coordinate options for spatial scatterer grids are possible: rectangular in $\left(x_{1},x_{2}\right)$ or polar in (d,α). In either case, prior knowledge can be used from the standard DRT processing to constrain the parameter span for the dictionary.

A rectangular scatterer grid was chosen spanning ±0.4 m with a nominal spacing proportional to λ/2. As for the standard DRT demonstration, the two frequencies of 8 GHz (λ=3.75 cm) and 12 GHz (λ=2.5 cm) are used. Over the selected discretization interval, the number $N_{\sigma }$ of atoms was 1849 (for 8 GHz) or 4096 (for 12 GHz).

**Figure 11:**
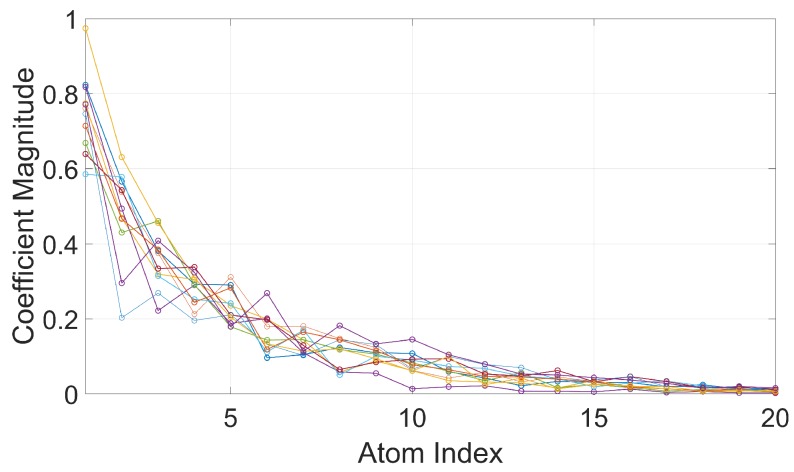
Magnitude of atom coefficients at f=8 GHz for first 20 atoms, shown for a subset of the total number of CPIs.

The coefficient magnitudes of the first 20 atoms extracted from the 8 GHz signal show a clear convergence, as shown in Figure 11.

To reduce signal processing noise effects in the resulting image, a simple thresholding method can be used to control the number of atoms to keep in the sparse representation: in each CPI, stop the OMP iteration when atom coefficient magnitude falls below 20% of the maximum magnitude, as an example. This criterion can also save on computational cost, as less atoms need to be extracted in the processing.

**Figure 12:**
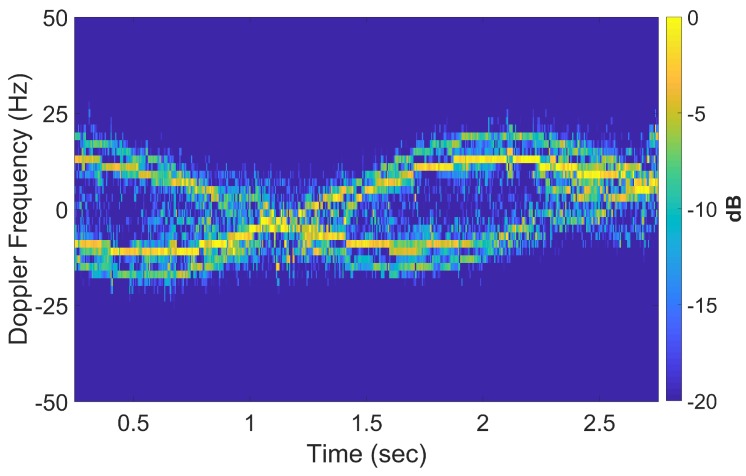
Spectrogram reconstructed, OMP-focused signal with first 20 atoms, at f=8 GHz (compared to Figure 7).

The spectrogram of the reconstructed and ‘OMP-focused’ signal is shown in Figure 12 which clearly shows more resolvable sinusoidal traces compared to Figure 7.

**Figure 13:**
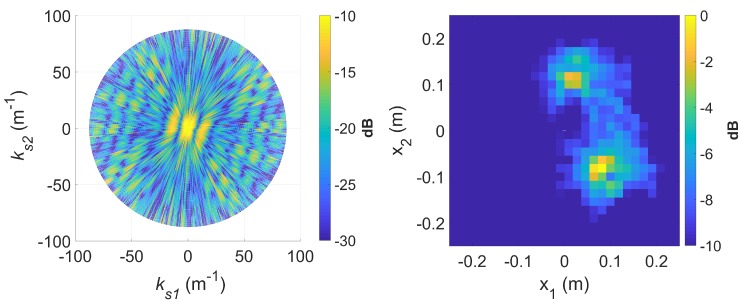
The slow time *k*-space support (left) and image (right) after OMP processing using a 20% coefficient magnitude threshold at f=8 GHz (compared to Figure 8).

An example is shown in Figure 13 for the 8 GHz signal. Compared to Figure 8, this is a clearly significantly more focused image where the scatterers are more easily resolvable.

**Figure 14:**
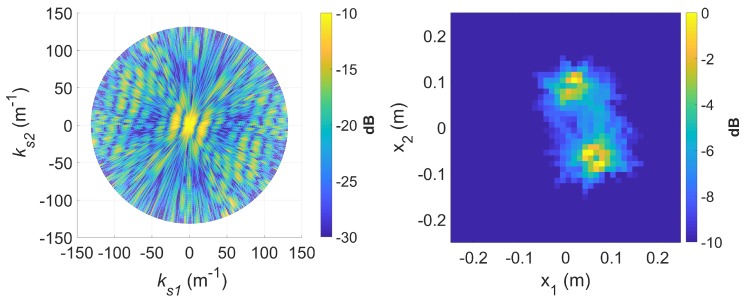
The slow time k-space support (left) and image (right) after OMP processing using a 20% coefficient magnitude threshold at f=12 GHz (compared to Figure 10).

For completeness, we also show results in Figure 14 for the 12 GHz signal, which also resolve the scatterers significantly better using the OMP processing as compared to Figure 10 for standard DRT. The double scattering effects resulting in ‘double rods’ are also enhanced.

### 4.2. Large Target

#### 4.2.1. Experimental Setup for Large Target

**Figure 15:**
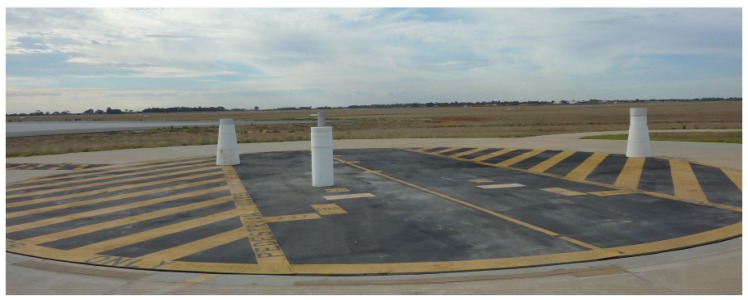
The turntable at the RAAF Edinburgh airbase with three metallic cylinders as a test target.

The experiment was carried out on the turntable at the RAAF Edinburgh airbase, which has a diameter of 17 m. The test target consists of three metallic cylinders as shown in Figure 15 with physical specification listed in Table 1. The experimental X-band radar employed a vertically polarised pulsed stepped frequency waveform starting at 9 GHz with 4 MHz steps, spanning a total of 256 frequencies. The turntable was rotated at approximately one revolution per 15 minutes with a receiver sampling rate of PRF=20 Hz at each frequency, which translates to an angular sampling interval of $0.02{}^{\circ }$.

#### 4.2.2. System Requirements

As previously described in Section 4.1.2 we designed the system requirements such that the backscattered radar signal is dependent on the angular sampling rate as follows.

- We choose $f_{k}=9$ GHz, corresponding to λ=3.0 cm. With $r_{\max}\approx 8.0$ m, $\Delta \theta_{DM}\approx 2.48{}^{\circ }$; this is well below the typical linear limit of several degrees. The system is thus $\Delta \theta_{DM}$-limited;
- $PRF_{a}=1067$ for this experiment which satisfies inequality (A1) for ambiguity free Doppler frequency.

The angular sampling interval of $0.02{}^{\circ }$ per sample in the experiment translates to a $PRF_{a}=2864$ samp/rad, which satisfies (A1).

Similar to the previous data set, an elliptic filter with a very narrow stop band is applied to the signal for clutter removal. Results are shown in Section 4.2.3 and Section 4.2.4.

#### 4.2.3. Standard DRT Imaging

**Figure 16:**
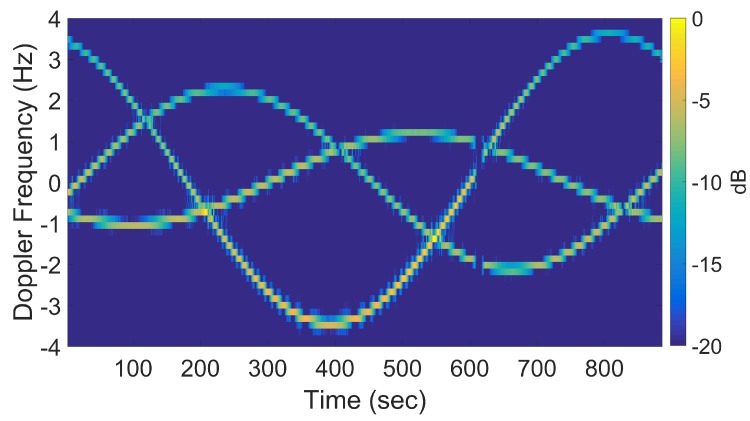
Spectrogram using standard DRT processing for f=9 GHz. (The small gap near 600 sec is due to an antenna pointing error during the measurements.).

**Figure 17:**
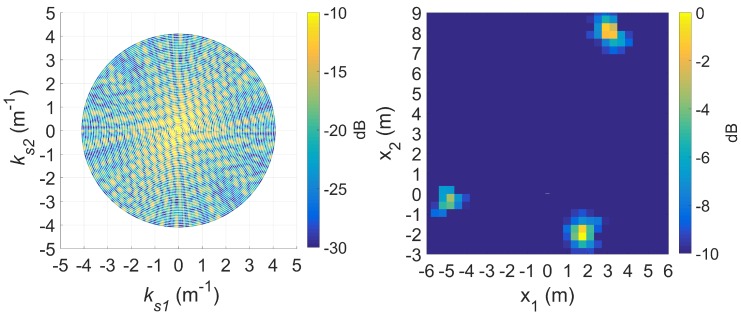
Slow-time *k*-space support (left) and image (right) for standard DRT processing; f=9 GHz.

Figure 16 shows a spectrogram of the signal at 9 GHz, featuring three distinct sinusoidal traces corresponding to the three cylinders. Figure 17 shows the corresponding slow-time *k*-space support and standard DRT image. The standard DRT imaging performance is poor due to the small diameter of the *k*-space support, with the cylinder locations represented by coarsely granulated pixels.

**Figure 18:**
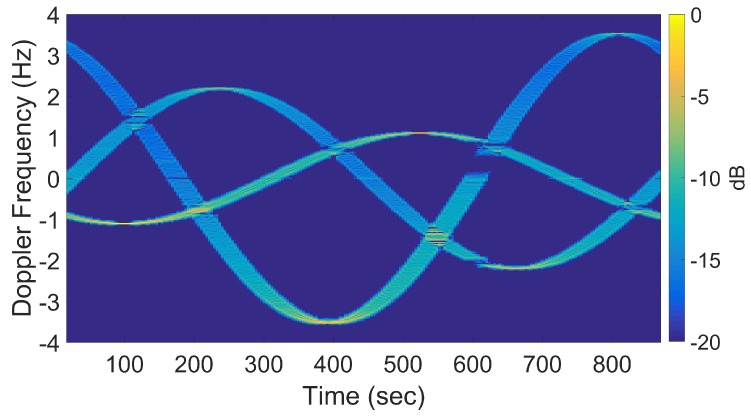
Signal spectrogram with augmented CPIs, κ=6 at f=9 GHz.

**Figure 19:**
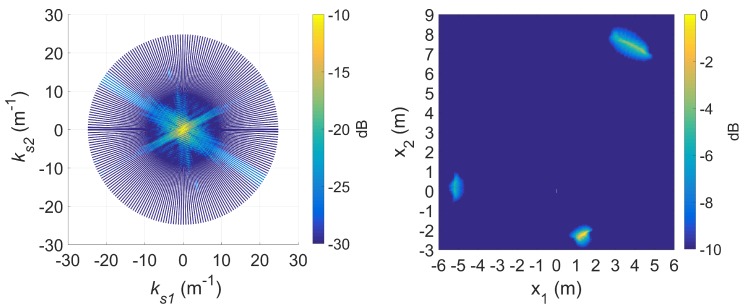
The slow time *k*-space support (left) and image (right) for standard DRT with κ=6, at f=9 GHz.

When longer CPIs are used with the standard DRT algorithms; for example, when κ in (16) is set to 6, the Doppler resolution in the spectrogram of the signal becomes higher, as evident in Figure 18; the corresponding cross-range profiles (with Doppler bin migration effects present) applied to standard DRT result in Figure 19.

Again it is shown that image blurring in standard DRT imaging is only in the azimuthal direction; image focusing is still generally achieved in the radial direction. The blurring effect in the image is more severe for scatterers at larger radial distances which travel along greater arc lengths within a given angular rotation angle (i.e., larger Doppler effects) and hence more severe Doppler bin migration.

#### 4.2.4. Augmented DRT Imaging with OMP

We choose the polar grids representation for this dataset as defined in Section 3.2.1. This approach is useful when some prior knowledge about the radial coordinate of the major scatterers is available from the standard DRT processing.

**Figure 20:**
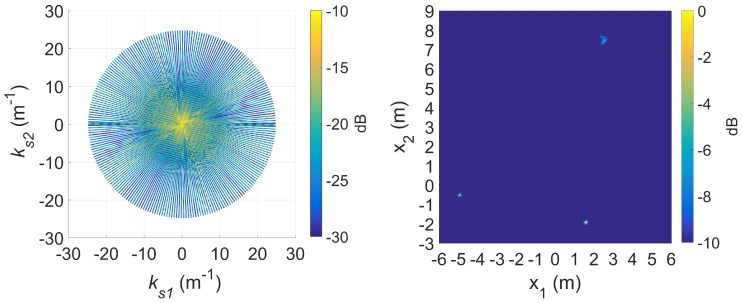
The slow time *k*-space support (left) and image (right) after OMP processing using a 20% coefficient magnitude threshold at 9 GHz (compared to Figure 19).

A relatively narrow window is used for discretization of the *d*-dimension derived from the Doppler information of the scatterers in Figure 16 with a λ/2 spacing. The full $360^{\circ }$ with $1^{\circ }$ spacing is used for α. Twenty atoms were extracted in each CPI from the OMP process giving a reconstructed spectrogram that is virtually identical to that in Figure 18, affirming the sufficient accuracy of the sparse representation. After the de-chirping operation, the scatterer locations are much more focussed as shown in Figure 20 compared to the same scatterers in Figure 19 with the same augmentation factor. The technique shows some degradation with the furthermost cylinder which exhibited the most blurring.

Our current study is focused more on imaging performance rather than computational cost; nevertheless, to give some idea on computational cost, we ran the algorithm on the high performance computer called ‘Phoenix’ at the University of Adelaide which took approximately 1 hour to run on 16 CPUs using 64 GB RAM.

## 5. Further Discussion

This paper is an expansion to the work reported earlier in [2], demonstrating high-resolution DRT imaging with real experimental data. As this is not a real moving and rotating target in a typical operational scenario, a number of issues could be noted.

Firstly, the target’s translational velocity is exactly zero for the entire data collection. Nevertheless, this is not expected to be a sensitive factor. For most real moving targets, translational velocity can be readily compensated by shifting the ‘body Doppler’ line to zero Doppler. Sensitive propagation phases, as in the case of fast-time *k*-spaces, do not enter the slow-time *k*-spaces.

Secondly, the measured data were collected at precise angular sampling rates $PRF_{a}$, which can only be estimated in typical operational scenarios. Errors in $PRF_{a}$ or $\Omega_{e}$ would translate into errors of the locations of populated samples as well as image scaling factor. Hence both image focusing and image scaling could be affected. We have not fully addressed these issues in this work.

The experimental data does reveal interesting electromagnetic phenomenology, highlighting the limiting simplicity of the ideal point-scatterer assumption; creeping waves and nonlinear scattering effects do exist, which are not taken into account in the current DRT theory.

On application of the OMP algorithm, what this work has demonstrated its feasibility: techniques such as OMP can be used for slow-time *k*-space augmentation. Other alternative sparse approximation techniques can possibly be used to yield higher performance. Numerous other aspects can also be considered, such as dictionary ‘learning’: how to select an optimum spatial scatterer grid for the best focusing performance while keeping computational cost at manageable levels? Or how to deal with the off-grid/mismatched scatterer problem [30]. Many open questions remain, some of which will be addressed in future publications.

## 6. Concluding Remarks

We have demonstrated, with two datasets, the ability to improve image resolution using a rotating target with an ultra-narrowband radar. The enabling signal processing technique presented was a combination of Doppler radar tomography and a sparse reconstruction technique such as OMP, with a unifying mathematical framework based on the slow-time *k*-space. We have shown that closely spaced scatterers can be resolved by illustrating the creeping wave effect when the scatterer size is similar to the radar wavelength. The technique also performed well addressing the adverse effect of blurring in the image with scatterers at larger radial distances to the centre of rotation. By compensating for the blurred scatterer locations in the image, the ability to resolve closely spaced scatterers is improved providing finer details for target recognition.

Although the demonstration of this technique is effective, the application to a real complex target with many non-ideal scatterers may present additional challenges including discontinuous scattering effects, larger dictionaries affecting computational cost and inaccuracies due to signal mismatch with finite dictionary elements. In future work, we aim at investigating the use of multiple widely separated radar receivers to reduce the requirement on large target rotation angles for DRT imaging, where the direct application of OMP may not scale efficiently for large amounts of data. The increase in data may require a modified approach such as dictionary learning to help reduce the computational cost.

## Figures and Tables

**Table 1:** Metallic cylinder configuration.

Cylinder	$r_{m}$ (m)	Diameter (m)	Height (m)
1	2.5	0.15	0.30
2	5	0.38	0.18
3	8	0.21	0.46

**Figure A1:**
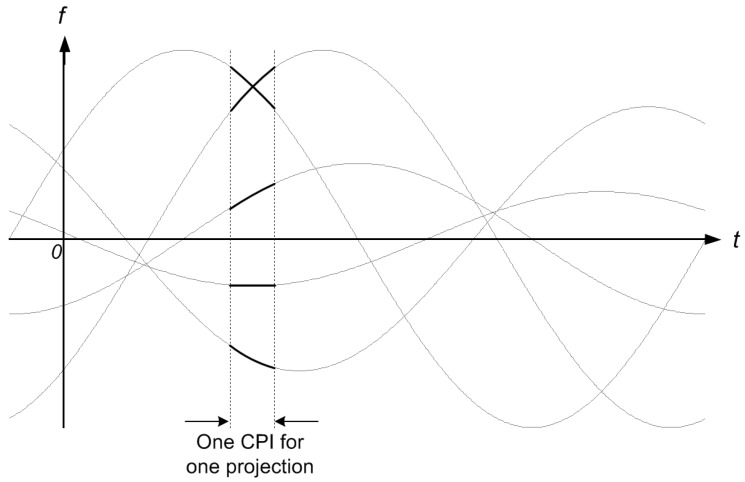
Instantaneous Doppler traces of point scatterers on a rotating target.

## Appendix A. Standard DRT: System Parameters and Image Resolution

Figure A1 illustrates the spectral composition of a segmented CPI in the DRT algorithm. The sinusoidal traces depicts the instantaneous Doppler frequencies of scattererers as the target rotates, which are generally chirp signals. The chirps are approximately linear for short CPIs.

There are three main constraints on system parameters for standard DRT to be applicable. The first one is Doppler ambiguity free condition: the sampling rate PRF must be at least two times the largest Doppler components in the received signal–the well-known Nyquist criterion. With $r_{\max}$ denoting a largest radial distance of scatterers on the target, this constraint can be written as

(A1) $$PRF\geq \frac{4\;\omega \;r_{\max}}{\lambda },\;\;\mathrm{or}\;\;PRF_{a}\geq \frac{4\;r_{\max}}{\lambda },$$

where $PRF_{a}=PRF/\omega$ is the angular sampling rate (in units of samples/rad).

The second constraint is: Doppler migration-free (DMF), i.e., variation of the instantaneous Doppler frequency of any scatterer is less than a Doppler bin size. This constraint may be derived as follows: a scatterer’s cross range is given by $x_{1}=r\;\cos\theta$, hence the differential change in $x_{1}$ is

$$dx_{1}=-r\omega \sin\theta \;dt.$$

Maximum cross range migration occurs near θ=nπ/2 for odd integral values of *n*. If dt represents a CPI time, here denoted as $T_{CPI}$, then the DMF requirement translates to having

(A2) $$|dx_{1}|\equiv r\omega T_{CPI}$$

to be not larger than a cross range bin size, at all range bins.

A cross range bin size $\Delta x_{1}$ is related to Doppler filter size Δf through the well-known relationship $\Delta f=\left(2\;\omega /\lambda \right)\Delta x_{1}$ for monostatic radars, hence

(A3) $$\Delta x_{1}=\frac{\lambda }{2\omega }\Delta f=\frac{\lambda }{2\omega }\frac{PRF}{K}.$$

Here, *K* denotes both the number of samples spanning the CPI and the FFT length; and therefore

(A4) $$T_{CPI}=\frac{K}{PRF}.$$

Using (A2), (A3) and (A4) in the DMF requirement $|dx_{1}|\leq \Delta x_{1}$ then leads to the condition

(A5) $$\Delta \theta =\frac{\omega \;K}{PRF}\leq {\left(\frac{\lambda }{2\;r_{\max}}\right)}^{1/2}\equiv \Delta \theta_{DM},$$

where Δθ is the (segmented) CPI rotation angle (or angular extent), and $\Delta \theta_{DM}$ is an effective angle the target would need to rotate to induce a Doppler migration (DM) through one frequency bin. Note that $\Delta \theta_{DM}$ depends only on radar wavelength λ and maximum radial extent $r_{\max}$, not rotation speed. Another useful related expression is

(A6) $$T_{CPI}=\frac{K}{PRF}\;\leq \;\frac{\Delta \theta_{DM}}{\omega },$$

for the corresponding CPI time.

The third constraint is the so-called ‘linear limit’: the maximum rotation angle at which the Taylor expansion in (6) up to the first order in time remains valid. In other words,

(A7) $$\Delta \theta <\Delta \theta_{LM},$$

where $\Delta \theta_{LM}\approx 10^{\circ }$.

Combining the three constraints above, the system constraints on PRF and *K* are (A1) and

(A8) $$K\leq PRF_{a}\;\min\left\{\Delta \theta_{DM},\;\Delta \theta_{LM}\right\}.$$

As an example, suppose $\Delta \theta_{LM}=8^{\circ }$ is used; and λ=5 cm, $r_{\max}=1$ m and ω=300 RPM, then (A5) gives $\Delta \theta_{DM}\approx 9.1^{\circ }>\Delta \theta_{LM}$, and the choice of PRF=15 kHz and K=64 would satisfy all constraints.

The result in (A8) also highlights a useful comparison between the DMF condition and the linear limit $\Delta \theta_{LM}$: the CPI length *K* may be limited by either of the two factors; it is however more desirable to be DMF-limited, i.e, without strong dependence on wide rotation angles. Indeed, shorter radar wavelengths and larger target dimensions would induce more pronounced Doppler effects which are required for the applicability of the DRT imaging algorithm itself.

Image resolution for standard DRT can be derived as follows. The minimum PRF for unambiguous Doppler effects, given by (A1), means a cross-range profile given by (11) exactly spans the cross-range extent of the target. Larger values would lead to outer range bins of the profile containing noise only, while those range bins spanning the target remain the same, in both bin size and number. For the case of minimum unambiguous PRF, the maximum selectable value of *K*, given by (A6), is

(A9) $$K_{STD}={\left(\frac{8\;r_{\max}}{\lambda }\right)}^{1/2},$$

or smaller if *K* is limited by $\Delta \theta_{LM}$ in (A8). The resolution of a cross-range profile is therefore

(A10) $$\Delta x_{RB}=\frac{2\;r_{\max}}{K_{STD}}={\left(\frac{\lambda \;r_{\max}}{2}\right)}^{1/2}.$$

The same result can be obtained from (10) and (7) by using the equality in (A5) for Δθ. The result in (A10) is also the expected image resolution in standard DRT imaging.
